# The impact of orthodontic treatment on the quality of life a systematic review

**DOI:** 10.1186/1472-6831-14-66

**Published:** 2014-06-10

**Authors:** Yu Zhou, Yi Wang, XiuYing Wang, Gerald Volière, RongDang Hu

**Affiliations:** 1 Department of Orthodontics, Hospital of Stomatology, Wenzhou Medical University, 113 west college road, 325000 Wenzhou, China; 2 School & Hospital of Stomatology, Wenzhou Medical University, Wenzhou, China

**Keywords:** Malocclusion, Orthodontic treatment, Quality of life, Oral health–related quality of life

## Abstract

**Background:**

Although a great number of specific quality of life measures have been developed to analyze the impact of wearing fixed appliances, there is still a paucity of systematic appraisal of the consequences of orthodontics on quality of life. To assess the current evidence of the relationship between orthodontic treatment and quality of life.

**Methods:**

Four electronic databases were searched for articles concerning the impact of orthodontic treatment on quality of life published between January 1960 and December 2013. Electronic searches were supplemented by manual searches and reference linkages. Eligible literature was reviewed and assessed by methodologic quality as well as by analytic results.

**Results:**

From 204 reviewed articles, 11 met the inclusion criteria and used standardized health related quality of life and orthodontic assessment measures. The majority of studies (7/11) were conducted among child/adolescent populations. Eight of the papers were categorized as level 1 or 2 evidence based on the criteria of the Oxford Centre for Evidence-Based Medicine. An observed association between quality of life and orthodontic treatment was generally detected irrespective of how they were assessed. However, the strength of the association could be described as modest at best. Key findings and future research considerations are described in the review.

**Conclusions:**

Findings of this review suggest that there is an association (albeit modest) between orthodontic treatment and quality of life. There is a need for further studies of their relationship, particularly studies that employ standardized assessment methods so that outcomes are uniform and thus amenable to meta-analysis.

## Background

Fixed orthodontic appliance therapy is a regular orthodontic treatment to correct variations from an arbitrary norm (align the teeth or correct other irregularities) [1], which may cause functional restrictions, discomfort and pain [2,3], but traditional orthodontic studies have only included clinician-based outcome measures.

Since Cohen and Jago [4] advocated development of ‘social-dental’ indicators, there has been considerable subjective patient-based measurement results leading to further understanding about psychosocial well-being and/or dental health [5]. Hence, oral health–related quality of life (OHRQoL) is defined as ‘the absence of negative impacts of oral conditions on social life and a positive sense of dentofacial self-confidence’. Thus, studying the OHRQoL index may provide information that will help clinicians and public health planners improve the quality of orthodontic care [6]. With regard to fixed orthodontic appliance therapy, understanding the consequences and discomforts during orthodontic procedures affords patients more realistic expectations regarding orthodontic treatment and may increase adherence to treatment [7,8].

Although many specific OHRQoL measures have been developed to analyse the impact of wearing a fixed appliance [9], there is still a paucity of systematic appraisal of the consequences of orthodontics on quality of life(QoL). QoL is important in providing an understanding of the importance of, and priority for, orthodontic care within the health care spectrum. Thus, the aim of this review was to assess the literature related to the impact of orthodontic treatment and orthodontic care on QoL, HRQoL and OHRQoL.

## Methods

To be included in the review, trials had to meet the following selection criteria:

Participants: patients receiving orthodontic treatment and non-orthodontic treatment

Interventions: Fixed or removable appliance, or interceptive orthodontic treatment.

Outcome measures: Changes in oral health–related quality of life (OHRQoL) from baseline (pre-treatment) to follow-up at least 1 month.

Exclusion criteria were lack of standardized measures in assessing QoL, HRQoL or OHRQoL; lack of effective statistical analyses; and case reports and review papers.

### Search strategy

PubMed, EMBASE, the Cochrane Central Register of Controlled Trials, and China Biology Medicine disc (CBM; to December 2013) databases were searched for relevant studies, up to and including December 2013.

There were no language restrictions. The search strategy is shown in Appendix 1. Reference lists of identified articles and relevant review articles were checked for further possible studies. Abstracts of all studies identified by the searches were independently assessed by two reviewers. Full copies of all relevant and potentially relevant studies or those with insufficient data in the title and abstract were obtained.

Information search on relevant journals (American Journal of Orthodontics and Dento-facial Orthopedics, European Journal of Orthodontics, Angle Orthodontist, Journal of Orthodontics, and World Journal of Orthodontics) was also performed. Unpublished literature was searched via Google-Scholar search engines and authors were contacted for further information where required.

### Assessment of relevance and eligibility

Titles and abstracts of studies identified in the search were screened by two reviewers, and full-text articles of relevant studies were obtained. Two reviewers independently assessed full-text articles for eligibility. Only studies which met all the eligibility criteria were finally included.

### Quality assessment and level of evidence

Papers included in the final review were assessed using the following parameters: (1) study design; (2) sample (source, sampling technique, sample size, and age characteristics); (3) assessment method of OHRQoL; (4) key findings and statistical inference(s); (5) level of scientific evidence based on the criteria of the Oxford Centre for Evidence-based Medicine [10], see Table 1.

**Table 1:** Oxford Centre for Evidence-based Medicine Levels of Evidence

**Level**	**Therapy/Prevention,aetiology/Harm**	**Prognosis**	**Diagnosis**	**Differential diagnosis/symptom prevalence study**	**Economic and decision analyses**
1a	SR (with homogeneity) of RCTs	SR (with homogeneity) of inception cohort studies; validated in different populations	SR (with homogeneity) of Level 1 diagnostic studies; with 1b studies from different clinical centres	SR (with homogeneity) of prospective cohort studies	SR (with homogeneity) of Level 1 economic studies
1b	Individual RCT (with narrow Confidence Interval)	Individual inception cohort study with > 80% follow-up; validated in a single population	Validating cohort study with good reference standards; or tested within one clinical centre	Prospective cohort study with good follow-up	Analysis based on clinically sensible costs or alternatives; systematic review(s) of the evidence; and including multi-way sensitivity analyses
1c	All or none	All or none case-series		All or none case-series	Absolute better-value or worse-value analyses
2a	SR (with homogeneity) of cohort studies	SR (with homogeneity) of either retrospective cohort studies or untreated control groups in RCTs	SR (with homogeneity) of Level >2 diagnostic studies	SR (with homogeneity) of 2b and better studies	SR (with homogeneity) of Level >2 economic studies
2b	Individual cohort study (including low quality RCT; e.g., <80% follow-up)	Retrospective cohort study or follow-up of untreated control patients in an RCT; Derivation of CDR or validated on split-samples only	Exploratory cohort study with good reference standards; after derivation, or validated only on split-samples or databases	Retrospective cohort study, or poor follow-up	Analysis based on clinically sensible costs or alternatives; limited review(s) of the evidence, or single studies; and including multi-way sensitivity analyses
2c	“Outcomes” Research; Ecological studies	“Outcomes” Research		Ecological studies	Audit or outcomes research
3a	SR (with homogeneity) of case-control studies		SR (with homogeneity) of 3b and better studies	SR (with homogeneity) of 3b and better studies	SR (with homogeneity) of 3b and better studies
3b	Individual Case-Control Study		Non-consecutive study; or without consistently applied reference standards	Non-consecutive cohort study, or very limited population	Analysis based on limited alternatives or costs, poor quality estimates of data, but including sensitivity analyses incorporating clinically sensible variations.
4	Case-series (and poor quality cohort and case-control studies)	Case-series (and poor quality prognostic cohort studies)	Case-control study, poor or non-independent reference standard	Case-series or superseded reference standards	Analysis with no sensitivity analysis
5	Expert opinion without explicit critical appraisal, or based on physiology, bench research or “first principles”	Expert opinion without explicit critical appraisal, or based on physiology, bench research or “first principles”	Expert opinion without explicit critical appraisal, or based on physiology, bench research or “first principles”	Expert opinion without explicit critical appraisal, or based on physiology, bench research or “first principles”	Expert opinion without explicit critical appraisal, or based on economic theory or “first principles”

Produced by Bob Phillips, Chris Ball, Dave Sackett, Doug Badenoch, Sharon Straus, Brian Haynes, Martin Dawes since November 1998. Updated by Jeremy Howick March 2009.

### Data extraction

Two reviewers independently screened all full-text articles and extracted data from the included studies using specially designed forms. Data were extracted on study design, characteristics of participants, characteristics of the intervention(s) and outcomes. Disagreements between the reviewers at the stages of eligibility-assessment, quality assessment and data-extraction, were resolved through discussion.

### Data synthesis

Pooling of data was based on study design, population characteristics, outcomes, OHRQoL-domains affected. Descriptive summaries of the studies were entered into tables and a narrative synthesis of evidence was planned.

## Result

**Figure 1:**
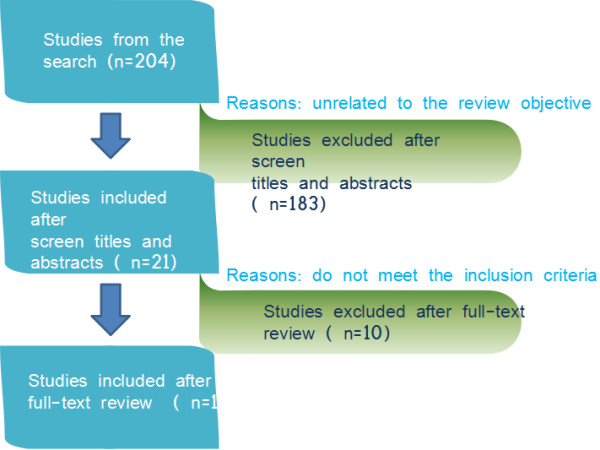
Flowchart for study inclusion and exclusion (n, number of studies).

A total of 204 potentially relevant articles were collected through initial literature and hand search, of which 183 were excluded based on their titles and abstracts. Full text articles of 21 studies were retrieved. Of these, 11 studies qualified for the review analysis. The flow of the inclusion process is shown in Figure 1.

Among the 11 included papers, six [11-16] were cohort studies with a good follow up and were classified as level 1B evidence according to the Oxford Centre for evidence based Medicine criteria. Four studies were cross-sectional designed, three [17-19] of which offered 3B evidence level because of the convenient samples; one [20] offered 2C evidence level due to the large community sample. The single remaining paper [21] was a case-control study with randomized sample type, which offered 3C evidence level.

Four QoL instruments, generic and oral health specific, were applied to the 11 included papers, namely OHIP, OIDP, CPQ and OHQoL-UK. OHIP (7 of 14) and CPQ (3 of 14) were the two most frequently used QoL instruments. OHIP was applied to both adults and Children, while CPQ was only used on children.

Given the fact that different methods were applied to measure QoL, and different time intervals employed in assessing QoL, meta-analysis was not feasible to combine these statistical results. However, a majority of studies indicated a correlation between orthodontic treatment and QoL no matter what measurement was applied. The cross-sectional studies revealed that orthodontic treatment resulted in a decrease in QoL scores. Whereas the longtitudinal studies indicated that with ongoing orthodontic treatment, the QoL score may increase a few weeks after initial orthodontic appliance placement. QoL might even be improved during the late stages of treatment (Table 2).

**Table 2:** Summary of Studies: Study Design, Sample and Quality-of-Life (QoL) Instruments

**Author**	**Study design**	**Sample type**	**Sample size**	**Age of sample**	**Length of follow-up time**	**Qol instrument**	**Univariaty analysis**		**Evidence level**
							**95% confidence intervals**	**P value**	
Eduardo, [21]	Case-control	Nonrandomized	837	15-16 y	Previous 6 month	CS-OIDP	0.16-0.46	p < 0.001	2C
Daniela, [14]	Longtitude	NOT mentioned	284	12-15 y	2 years	OHIP-14	—	p < 0.01	1B
Shoroog, 2011	Longtitude	Consecutive	118	11-14 y	More than 2 years	CPQ11-14	—	p < 0.01	1B
Zhijian, [15]	Longtitude	Consecutive	232	16 and older	18 month	OHIP-14 and OHQoL-UK	—	p < 0.01 (6 m), P < 0.01 (12 m), p < 0.05 (18 m)	1B
Man, [13]	Longtitude	Consecutive	198	13.1 ± 1.5	6 month	CPQ	—	p < 0.001 (first week), P < 0.05 (1 month)	1B
Mu, 2009	Longtitude	NOT mentioned	250	Mean 15, 7 y	More than 2 years	OHIP-14	—	p < 0.001	1B
Yueming, 2012	Longtitude	Consecutive	300	18-36 y	From begin to 6 months after posttreatment	OHIP-14	—	p < 0.01	1B
Andrea, 2011	Cross-sectional	Nonrandomized	579	11-14 y	-	CPQ11-14	1.11-2.33	p = 0.007	3B
Aihua, [19]	Cross-sectional	Nonrandomized	182	7-33 y	1-month post-insertion	OHIP-14	—	p < 0.01	3B
Nathalia, 2012	Cross-sectional	Consecutive	200	18-30 y	After treatment more than 6 month	OHIP-14	—	p < 0.01	3B
Oliveira, 2003	Cross-sectional	Nonrandomized	1675	15-16 y	-	OIDP and OHIP	1.30-2.62	p < 0.001	2C

## Discussion

The present review was performed to systematically analyze the impact of orthodontic treatment on quality of life. The results suggest that there is an association between orthodontic treatment and QoL. Most studies reported that patients were considerably compromised in terms of their overall OHRQoL until approximately 1 month after appliance insertion. The severity of the compromised condition in terms of overall OHRQoL was greatest at 1 week with the reported impact on physical pain, psychological discomfort, and physical disability. Patients’ OHRQoL was better after they completed orthodontic treatment than it was before or during treatment. Only one study [17] reported worse OHRQoL compared with a control group with no malocclusion and not wearing a fixed appliance.

QoL is a somewhat intangible entity and there has been much debate as to how to define it. However, since there is general consensus that QoL reflects physical, social, and psychologic functioning, these terms formed the basis of the literature search methodology [22]. It is now generally accepted that the measurement of oral health-related quality of life is an essential component of oral health surveys, clinical trials, and studies evaluating the outcomes of preventive and therapeutic programs intended to improve oral health. The assessment of oral health-related quality of life has an important role in clinical practice. There were a number of studies measuring oral health-related quality of life. The literature search yielded more than 200 potentially relevant articles, demonstrating a paradigm shift from the biophysical focus of malocclusion to a more patient-centered focus of orthodontic treatment and management. Furthermore, it was apparent that QoL had been a particularly common topic of research in the past decade within all dental disciplines [23].

In this review, a recommended approach to the conduct, methodology and reporting of systematic reviews was followed [24]. Only studies which used previously validated measures of OHRQoL were included. Finally, there were 4 instruments measuring oral health-related quality of life in this study. The Child Perception Questionnaire (CPQ) and the Oral Health Impacts Profile (OHIP) measure were most frequently used in the assessment of OHRQoL. This shows that there is no shortage of standardized assessment methods for documenting changes in OHRQoL. However, the use of different assessment methods makes it difficult to conduct a meta-analysis.

Perhaps not too surprisingly, the majority of the research in this area has focused on the impact of orthodontic treatment on the QoL in children rather than adults. This relates in part to the fact that children make up the majority of orthodontic patients, although it is increasingly recognized that more and more adults are seeking correction of their malocclusion [25].

In this systematic review, we included both short (3/11) and long-term follow-up (6/11) studies so as to assess changes during and after orthodontic treatment. From the short-term results, Man [13] found that changes in OHRQoL occur after fixed orthodontic appliance therapy. Compared with pretreatment, a patient’s OHRQoL is frequently worse during treatment (oral symptoms, functional limitations), although it is better in some aspects (emotional well-being). The period of greatest change in OHRQoL occurs during the first month of treatment. Ling Aihua [19] and Eduardo [21] found similar results indicating that orthodontic treatment does affect patients' OHRQOL, the impact being more serious in the first month of treatment. The longitudinal data [7,11,14-16,18] on pre- and post-orthodontic treatment showed that both children and adult patients who received orthodontics had significantly better oral health-related quality of life scores in the retention phase, after treatment completion, compared to non-treated subjects. Zhijian [15] also found that the greatest deterioration in OHRQoL occurs in the early phase of treatment; the detrimental effects on OHRQoL are reduced with ongoing treatment. Daniela [14] found that fixed orthodontic treatment in Brazilian children resulted in significantly improved OHQoL after completing 2 years of orthodontic therapy.

The level or strength of evidence that can be gleaned from the included papers was relatively high. Most were longitudinal studies with good follow up, despite lacking randomized controlled trials (RCTs). Because of ethical issues, orthodontics frequently does not lend itself very well to randomized controlled trials, particularly when children are involved. Of note, studies generally observed an association between orthodontic treatment and HRQoL, irrespective of how the parameters were assessed. However, the inferences from the correlation statistics and regression findings would indicate that the strength of the association could be interpreted as moderate.

Certain limitations must be acknowledged. Most of the included studies were observational and therefore at a greater risk of bias and confounding which could compromise the internal and external validity of their results [26].

Many factors, such as age, gender and psychological and socio-economic factors might have confounded the outcomes of treatments and patients’ perceptions of QoL. Furthermore, different OHRQoL measures were used in the included studies, potentially leading to differences in the way OHRQoL was assessed, as certain measures are more likely to capture specific aspects (domains) of OHRQoL than others.

Attention also should be paid to the potential possibility of publication bias, not only are positive results more easily published, but it is also difficult to retrieve all the available literature. The results are vulnerable to the impact of publication bias, which may be minimized by obtaining printed copies of orthodontic journals, and prone to the limitations of accessing online data references, or conducting Google or other electronic searches.

Finally, a meta-analysis was not performed because of concerns that the clinical and methodological diversity (heterogeneity) of the included studies would make the process meaningless. Studies differed in (1) participants; (2) the outcome (OHRQoL) measures used and (3) durations of follow-up. Confounding factors and potential biases in the data might have further compromised a meta-analysis leading to a misinterpretation of results.

Therefore, future research should be improved as follows: First, by adopting a common OHRQoL-measure to ensure consistency of recording outcomes and enable meta-analysis of their relationship. Second, further well-designed prospective cohort studies with long-term follow-up are required to confirm and replicate the findings reported here. Future RCTs should evaluate OHRQoL-outcomes in patients receiving orthodontic treatment, while adjusting potential confounding factors. The impact of therapy on OHRQoL in patients with varying severities of malocclusion also requires investigation.

## Conclusions

In literature there is a growing interest in the relationship between orthodontic treatment and HRQoL and it suggests that orthodontics can moderately improve the OHRQoL of patients. In future, however, there is still a need to determine appropriate assessment methods of orthodontic treatment and of quality of life (QoL, HRQoL, and/orOHRQoL measures) to enable meta-analysis of their relationship.

## Appendix 1

Search strategy for pubmed

#1 (“orthodontic”[Mesh]) OR (orthodontics)

#2 (orthodontic AND (therapy OR treatment))

#3 (quality of life OR oral health related quality of life OR QoL OR OHRQoL)

#4 (impact AND (orthodontic AND

(therapy OR treatment)))

#5 (#1 AND #2)

#6 (#5 OR #2)

#7 (#3 OR #4)

#8 (#6 AND #7)

## Competing interests

The authors declare that they have no competing interests. There is no support and funding source for conducting the review.

## Authors’ contributions

YZ and RDH designed the study, gathered the information, performed the statistical analysis and wrote the first draft of the manuscript. YW and XYW designed the form for data gathering and supervised the statistical analysis. GV reviewed and translated the manuscript. All authors read and approved the final manuscript.

## Pre-publication history

The pre-publication history for this paper can be accessed here:

http://www.biomedcentral.com/1472-6831/14/66/prepub
